# A globally networked hybrid approach to public health capacity training for maternal health professionals in low and middle income countries

**DOI:** 10.1186/s41256-017-0027-x

**Published:** 2017-03-01

**Authors:** Scott McIntosh, José G. Pérez-Ramos, Tamala David, Margaret M. Demment, Esteban Avendaño, Deborah J. Ossip, Timothy De Ver Dye

**Affiliations:** 1 Department of Public Health Sciences, University of Rochester Medical Center, 265 Crittenden Blvd., CU 420644, Rochester, NY 14642 USA; 2 Department of Obstetrics and Gynecology, University of Rochester Medical Center, Rochester, USA; 3 Department of Nursing, the College at Brockport, State University of New York, Rochester, NY USA; 4 University of Rochester School of Nursing (URSON), Rochester, NY USA; 5 Department of Obstetrics & Gynecology - University of Rochester Medical Center, Rochester, NY USA; 6 UCIMED, San José, Costa Rica

**Keywords:** Maternal Health, Community Engagement, Dominican Republic, Learning Management System, Online Training

## Abstract

**Background:**

MundoComm is a current NIH-funded project for sustainable public health capacity building in community engagement and technological advances aimed at improving maternal health issues. Two to four teams are selected annually, each consisting of three healthcare professionals and one technical person from specific low and middle income countries (LMICs) including Costa Rica, Dominican Republic, Honduras, and other LMICs. MundoComm is a course with three parts: in-person workshops, online modules, and mentored community engagement development. Two annual 1-week on-site “short courses” convened in Costa Rica are supplemented with six monthly online training modules using the Moodle® online platform for e-learning, and mentored project development. The year-long course comprises over 20 topics divided into the six modules - each module further segmented into 4 week-long assignments, with readings and assigned tasks covering different aspects of community-engaged interventions. The content is peer reviewed by experts in the respective fields from University of Rochester, UCIMED in Costa Rica, and faculty from Costa Rica and the Dominican Republic who maintain regular contact with the trainees to mentor learning and project progress. The purpose of this paper is to report the first year results of the MundoComm project.

**Methods:**

Both quantitative and qualitative feedback (using online data capturing forms) assess baseline and post-training knowledge and skills in public health project strategies.

**Results:**

The course currently has one team each in Costa Rica, the Dominican Republic, and Honduras for a total of 12 trainees. The course and modules include best practices in information and communication technologies (ICTs), ethical reviews, community engagement, evidence-based community interventions, and e-Health strategies. To maximize successful and culturally appropriate training approaches, the multi-media didactic presentations, flexible distance learning strategies, and the use of tablets for offline data collection are offered to trainees, and then feedback from trainees and other lessons learned aid in the refinement of subsequent curricular improvements.

**Conclusions:**

Through remark and discussion, the authors report on 1) the feasibility of using a globally networked learning environment (GNLE) plus workshop approach to public health capacity training and 2) the capacity of LMIC teams to complete the MundoComm trainings and produce ICT-based interventions to address a maternal health issue in their respective regions.

## Background

In many low and middle income countries (LMICs) where health is improving more slowly compared to wealthier countries, international health disparities increase. Improvements in child and maternal mortality, for example, have stagnated in several countries and, although a trained workforce of health professionals is essential to successfully address this, there is currently a lack of adequate capacity [1].

Costa Rica, which has emerged as a regional hub for technology and health in Latin America [2, 3] serves as the setting for on-site training and host of the online training modules for the current project. This training initiative, called MundoComm, is funded by the National Institutes of Health (NIH) and links a US-based project team with an expanded team of informatics faculty that includes Fellows/Trainees from our previous projects in Costa Rica and the Dominican Republic [4–6]. With this experienced partnership team, the aims of MundoComm are to train and demonstrate the successful use of Information and Communications Technology (ICT) for maternal health improvement throughout the Latin American region. Despite progress in other areas of maternal and child health, this region faces ongoing poor profiles of maternal mortality rates [7].

Elsewhere such technological innovations as m-Health and internet-based technologies have improved maternal health [8, 9]. Our partners in Costa Rica, the Dominican Republic and in other areas of Latin America (e.g., Honduras, Mexico) are eager to demonstrate feasibility of such technological innovations for specific populations and regions where maternal health is challenged, particularly in the indigenous communities, rural communities, and among the African-Caribbean population.

MundoComm’s primary aims are:

1. To develop a mentored training program for community-based public health applied research teams from high-need areas of Costa Rica, the Dominican Republic, Honduras and elsewhere in Latin America to learn about ICT applications, with specific reference to maternal health;
2. To test specific training team ICT innovations in field settings to demonstrate feasibility and appropriateness for local context;
3. To create a “Collaboratory” environment where teams, faculty, experts, and others to share developing material, comment on plans, suggest alterations and developments, discuss barriers, and provide support for trainees in the innovation process; and,
4. To stimulate development of a professional network of ICT, medical, public health, and community students, faculty, researchers, and practitioners devoted to supporting ICT for Maternal Health in Latin America.

The theoretical orientation of MundoComm blends Team Learning Theory (TLT) [10, 11] with Community-Engaged Research (CER) [11, 12] to create a mentored, experiential training year where local teams of professionals develop an ICT innovation for their area and context. Both TLT and CER directly contribute to the lasting sustainable effects of MundoComm. Team-based learning and the community-engagement process invests in groups of people, with distinct but overlapping roles, so that there is common exposure and group experience that promotes institutional memory, cross-training, and succession planning [13, 14]. Should a team member leave, teams can take on new members without requiring new members to repeat the entire training, rather their induction can involve completing the online modules and reviewing archival materials, with mentoring provided by other team members.

The objective of the present analysis is to explore and report upon the first year results of the MundoComm project. GNLEs (globally networked learning environments) are highly goal-based learning environments that incorporate innovation and technological change. The feasibility of combining a GNLE and workshop approach to public health capacity training, and the capacity of LMIC teams to complete the Mundocomm trainings and produce ICT-based interventions to address a maternal health issue in their respective regions is useful not only to the current project team, but hopefully exportable to other project teams in other LMIC settings. The University of Rochester’s Research Subjects Review Board reviewed the project and determined that the project does not qualify as human subjects research (45 CFR 46.102) in that the activities do not meet the federal definition of research.

## Methods

### The MundoComm project

The aims of MundoComm are to create a “Collaboratory” [15] – with both online and in-person occasions for interaction, mentoring, sharing of technology, and the expansion of existing distance-learning resources. Through MundoComm, the partnerships and areas of expertise aims to test, evaluate, and provide integrated technological and public health training simultaneously through in-person and online methods to local teams in high-risk areas with multiple professional roles represented (“integrated teams”). By training multiple people across role types from different communities, the project aims to maximize chances of impacting institutional sustainability, and assuring community engagement.

### The MundoComm curriculum

In the Collaboratory context described above, MundoComm is implementing 1) workshops, 2) online training, and 3) a mentored community training. Two week-long short courses each year are provided to address intervention development and evaluation, health registry development, technology assessment, and ICT project management. These are presented to project teams in-person at the Costa Rica site, and ultimately made available to remote and passive users throughout Latin America. The short courses are supplemented by monthly online training modules (described below). Finally, team mentors meet regularly with the project teams to continually improve program infrastructure and resources.

### Participants

Two to four integrated teams per year, each consisting of three healthcare professionals and one technical (e.g., IT, statistical) person from a specific Low and Middle Income Country (LMIC), will be trained annually in the development and conduct of community engaged projects aimed at improving locally determined Maternal Health issues in their respective countries (Costa Rica, Dominican Republic, Honduras, and other LMICs over a 3-year training period).

In the first year, the course had 12 enrolled trainees including one team each from Costa Rica, the Dominican Republic, and Honduras. The cohort was composed of nurses, physicians, licensed engineers (technology) and masters’ level hospital administrators. As required for the integrated team approach, each team had three healthcare professionals (including at least one physician), and one IT specialist. The personnel, country, and proposed project are listed in Table 1.

**Table 1:** Proposed Projects

Country Team	Trainees	Proposed Project using TICS
Costa Rica	3 physicians, 1 IT specialists	Evaluate impact of previous TICS projects on maternal and child health in rural community
Honduras	1 physician, 1 administrator, 1 nurse, 1 IT specialist	Training in rural teen pregnancy prevention and post-partum follow-up
Dominican Republic	2 physicians, 1 administrator, 1 IT specialist	Lower risk of premature births and assess effects of poor nutrition, toxemia, and post-partum complications in low-resource areas

### Trainers and mentors

The Universidad de Ciencias Médicas (UCIMED), a large medical School in San José, serves as the primary academic base for MundoComm. UCIMED has an active distance-learning portal for continuing education and is especially active in community-engaged service projects. Trainers and mentors included doctoral-prepared faculty from UCIMED, Costa Rica, the Dominican Republic and the University of Rochester. Faculty from Costa Rica and the Dominican Republic speak Spanish as their primary language, and included physicians with additional post-graduate training in community and public health, informatics, and/or clinical investigation. While faculty and mentors from the University of Rochester were not bilingual (except for one who is originally from Puerto Rico), all trainings involving non-Spanish speakers and all English materials were presented with Spanish interpretation. University of Rochester faculty disciplines included: Anthropology, Nursing, Psychology, and Informatics.

## Technology

### Online training platform

Moodle® (Modular Object-Oriented Dynamic Learning Environment), a freely available and popular open-source learning management system (LMS) or e-Learning platform that serves educators and learners, was developed in 2002 to help educators create online courses with a focus on interaction and collaborative construction of content [16–18]. The feasibility of using a such a globally networked learning environment (GNLE) is central to MundoComm’s overall goals.

MundoComm trainees were asked to create names for their respective teams. This was implemented: 1) to promote the identity, cohesiveness, and effectiveness of each field team, and 2) to promote efficient and tailored faculty-team communication via the LMS. Four named groups were created within this platform, and the course faculty manually enrolled each trainee into his or her respective group. After trainees submitted their completed assignments online, course faculty were able to provide evaluation, instruction, and feedback specific for each team. Additional team-based organization regarding the timing and management of assignments was accomplished by controlling the visibility/accessibility of specific modules, comments, and postings.

### REDCap data collection and management

In tandem with the Moodle® platform, online didactic assignments and evaluations were developed and implemented using REDCap® (Research Electronic Data Capture), which is a software toolset and workflow methodology for electronic collection and management of research and clinical trial data with a secure online format providing an easy-to-use interface for trainees to enter requested information and to provide feedback to the course faculty.

The Clinical Translational Science Institute (CTSI) Informatics Core, a unit of the University of Rochester School of Medicine and Dentistry’s Academic Information Technology (AIT) Group, serves as a central facilitator for all data management, including project-specific data and iterative self-documenting processes by all members of the project team. REDCap® was developed in a manner consistent with HIPAA security requirements.

### Modules and materials

#### Spanish back translation

In order to create an equivalent and culturally appropriate Spanish language version of initial English versions, materials were first translated into Spanish, and then back translated into English using the Brislin method [19]. The Spanish versions were pre-tested during the back translation process by four bilingual investigators from the US and Costa Rica for readability, skip patterns, formatting, and content.

## The course

Learning Objectives for the course are to: 1) provide the didactic and mentoring infrastructure to facilitate ICT innovations developed and tested for feasibility in community-based real-life settings; 2) infuse each institution and each of the trainee teams with new insights and strategies for working with diverse partners to create ICTs; 3) aid the teams and faculty to improve the efficiency of the development process via the Collaboratory concept of mentoring and social networking; and 4) strengthen the academic collaboration amongst the partners.

**Fig. 1:**
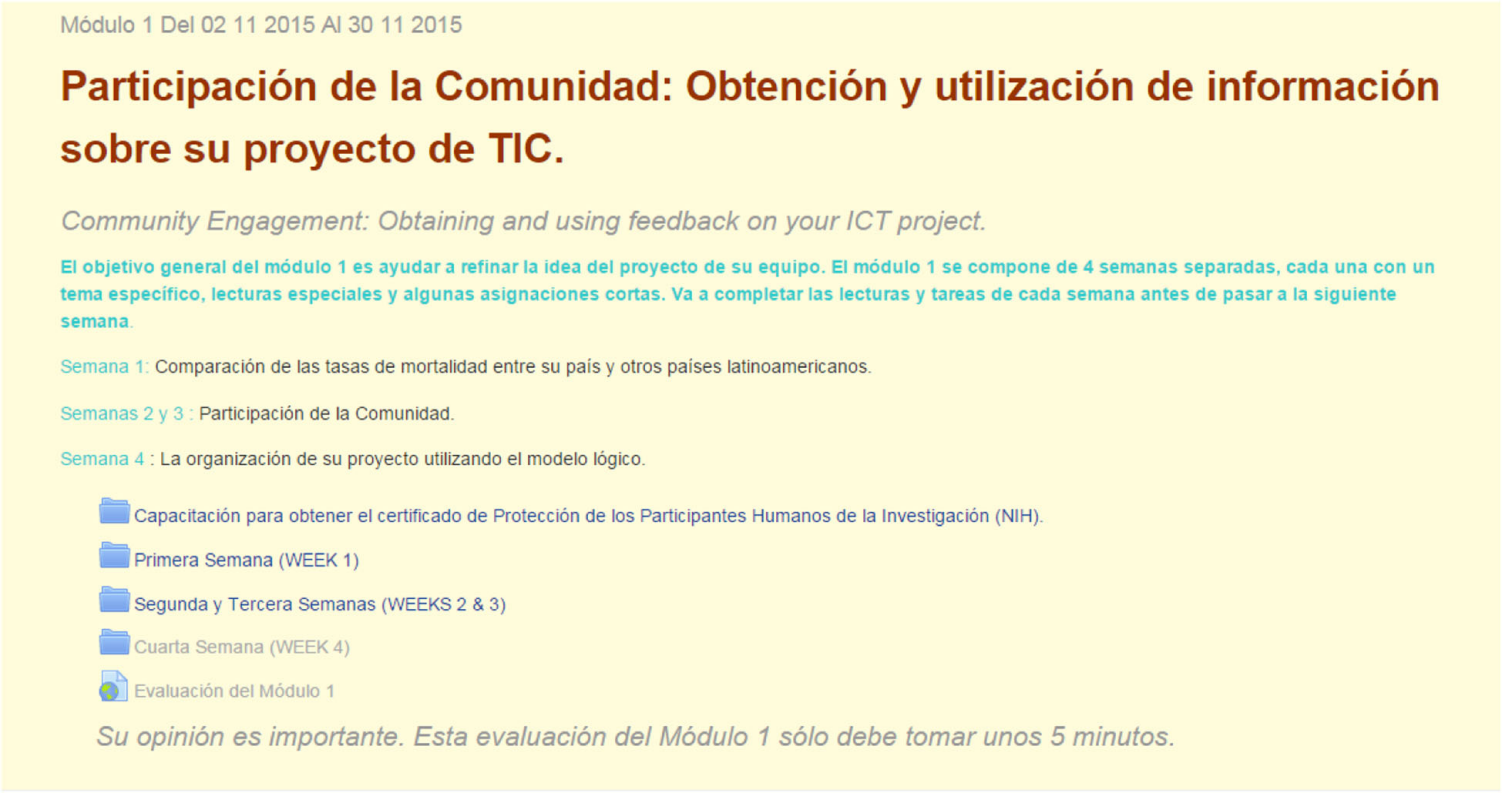
Screen Shot: Online Module #1

To address these Learning Objectives, a core educational plan over a 12-month timeframe was developed (Table 2) which includes two annual 1-week courses convened in Costa Rica (Table 3), and further supplemented with six online training modules, using the Moodle® online training platform and mentored guidance. The online course comprises over 20 topics divided into the six modules - each module further segmented into three-four assignments, with each assignment being 1 to 2 weeks in duration. Assignments include readings and assigned tasks covering different aspects of community-engaged public health interventions. The content is peer reviewed by experts in the respective fields from University of Rochester, UCIMED in Costa Rica, and faculty from Costa Rica and the Dominican Republic who maintain regular contact with the trainees to mentor learning and project progress (Fig. 1). Attention was given throughout the short-course and online modules to best-practice recommendations for data management and security, and well as ethical considerations in the conduct of research. All participants completed an NIH ethics course as part of their training.

**Table 2:** Core Educational Plan

	Application	1	2	3	4	s	6	7	8	9	10	11	12	Beyond
Team ICT Project	Team and ICT resources identified; general ICT protect topic identified; assets assessment completed in application	Team Completes ICT Preliminary Project Planning Worksheet	Teem finalizes ICT project plan at workshop	Team -implements ICT project plan; Completes Monthly Protect Update	Team implements ICT protect plan	Team implements ICT project plan	Prototype Presentation online	Prototype revision based on feedback	Final Prototype Revision	Workshop in San Jose to modify and finalize prefect plans	Feasibility Testing	Final revisions based on workshop feedback	Final Prototype Presentation Online	Team works toward further development, testing, and deployment accessing project resources as needed
ICT Training Modules	Documents learning resources and tene commitment in application	Team completes preparatory module on-line ICT for Maternal Health	Team participates in week-long Short Course I/workshop m San Jose	Team completes on line module “Obtaining and Using Feedback on Your ICT Project”	Team completes online module “Creating a Social Media Presence for your ICT Project”	Team completes on-line module on’ Crowdsourcing ICT Ideas	Team completes module “Presenting Your ICT Prototype”	No online module (Technical work)	Team completes online module “Moving ICT Project from Development to Testing”	Short Course II (In-person) No online module	Complete online module “ICT Evaluation, Measures, and Statistics”	Complete online module “Preparing your Final ICT Project Prototype”	Team completes online module “After prototyping: Planning for ICT Project Deployment”	Complete revised modules as needed
Team Mentoring/Networking	Team provides Letter of Recommendation on from community or institution	Start-up Collaboratory Call between Mentors and Team	In-person Mentoring and Consultation at Workshop in San Jose	Hold a community/institutional discussion about project to obtain feedback	Team designs its project’s social network page	Team crowdsources prototype ideas	Obtain feedback - crowdsource and social network	Iteratively share revisions for feedback	Iteratively share revisions for feedback	Test products and ideas with other trainee colleagues	Work with at least one community site to test product	Gather user feedback from test site(s)	Obtain feedback from social networking site	Expansion of network; Continued use of mentors as needed; Evaluation Visits; Symposium participation
Ongoing	Internal consultative process with other local stakeholders	Weekly blog updates												
		Bi-weekly call with mentors												
		Access consultation resources ad-hoc												
		Crowdsource development ideas and problem solve												
		Completes Monthly Protect Update												
Focus		Planning, Brainstorming, Ideation, Impact	Planning, Logistics, lICT Areas	Technical Development	Social Networking	Crowdsourcing/Community- Engaged Design	Communicating	Revising	Testing	Impact	Evaluation	Dissemination	Spin-off and dose-out	Networking, sustained involvement

**Table 3:** Sample Schedules for ICT in Maternal Health Short Courses I and II

Short Course I				
Day 1	Day 2	Day 3	Day 4	Day 5
Introduction and Short Course Evaluation Intake	Smart Databases and Registries: REDCap Basics	Web applications: WordPress and HTML Basics	Cloud Computing: OwnClowd, Google Cloud, and Amazon Web Services Basics	Team presentations of ICT project
ICT in Maternal Health: What’s the Evidence	Smart Databases and Registries: i2b2 workshop	Web applications: WordPress and HTML workshop	Social networking: Facebook, Twitter, LinkedIn, Google Analytics	Feedback from mentors/participants
Working lunch: Meet the Faculty/Brainstorming	Working lunch: Develop a logic model for maternal health problem	Working lunch: Create an ICT product plan	Working lunch: Refine your plans	Working lunch: ICT and Community Engagement
Social, Legal, and Ethical Considerations in ICT	mHealth applications: PhoneGap and OpenMEAP Basics	Analytics: EpiInfo Basics	Sustainability of ICT and public health projects	ICT in Maternal Health, Revisited
Facilitated completion of Citi Program/Ethics Course in Spanish	mHealth applications: PhoneGap and OpenMEAP workshop	Workshop with Mentors	Site visit to Cenfotec Labs	Short Course Evaluation/Closeout
Short Course II				
Day 1	Day 2	Day 3	Day 4	Day 5
Introduction and Short Course Evaluation Intake	Implementing ICT Interventions	Lab workshop with mentors	Advanced Communications technologies	What comes next? Team Presentations
Taking Stock: Where are you now? Team Presentations	ICT Research in Communities	Lab workshop with mentors	Creating a Community of Practice: ICT for Maternal Health	Feedback from mentors/participants
Lunch ICT Presentation	Lunch ICT Presentation	Lunch ICT Presentation	Lunch ICT Presentation	Lunch ICT Presentation
ICT Usability Assessment	Finding Funding for ICT I: Grants and Contracts	ICT Training Strategies	Global Health ICT Resources	Meeting with Ministries of Health and Technology
ICT Usability Assessment Workshop	Finding Funding for ICT II: Crowdfunding	Workshop with Mentors	ICT in Maternal Health: Evaluation	Short Course Evaluation/Closeout

### ICT in maternal health I short course: technology

The content areas of instruction identified for this population of medical learners include introductory-level skills and topics designed to train health and IT professionals for whom research is not a central job responsibility, but for whom understanding and basic implementation of research principles may help them to be more successful with their maternal health project development. The course was designed to help trainees be more knowledgeable and effective in developing novel technology-based interventions to serve their maternal health populations; to help them understand their project’s data needs and to successfully organize their project from inception to pilot testing.

During the first week-long short course training in Costa Rica with teams from the Dominican Republic, Honduras and Costa Rica, didactic material was presented in person by United States, Costa Rica, and Dominican Republic faculty covering a wide range of introductory-level areas. The Introductory-level areas of instruction were identified from inspection of methodology courses offered on various online learning platforms, and input from content experts on the core project team, and colleagues within their professional networks. The content areas were then organized into overarching themes of instruction and more specific topics to be addressed via modules, as shown below, and placed either in the in vivo short-course format, or the online distance-learning platform (Moodle®). These themes were identified with the goal of providing *an introductory experience*, as appropriate for non-research professionals.

Overarching Themes: 1-Week Short Course (See Also Table 3)

1. Research and ICTs (Information and Communication Technologies) in Latin America.
2. ICTs in Maternal Health
3. Aspects of social, legal and ethical considerations in ICT.
4. Facilitated Global Health Ethics: bioethics program in Spanish.
5. Intelligent databases and data records.
6. Intelligent databases and data records: workshop with Google® Forms.
7. Development of a logic model to address a problem of maternal health.
8. Clinical research and databases.
9. Basics: Epi-Info®
10. How to create a plan for development of an ICT product.
11. Review and analysis tools for the evaluation and accompaniment of projects from the first year of MundoComm
12. Cloud (cloud) computing: OwnCloud®, Google Cloud® and Amazon Web Services® (AWS®).
13. Introductory to Word Press® and HTML.
14. Resetting and revision of work plans.
15. Other tools: Open MEAP®, Phone GAP®.
16. Use of social networks and community empowerment: Facebook®, Twitter®, LinkedIn®, Google Analytics®.
17. The sustainability of projects and ICT.

Overarching Themes: Online Training (See Also Table 3)

1. Module 1: Community Engagement
2. Module 2: Social Media & ICT Project
3. Module 3: Crowdsourcing ICT Prototype
4. Module 4: From development to testing
5. Module 5: ICT Evaluation, Measures and Statistics
6. Module 6: Final ICT Prototype

#### Guided mentoring

ICT technologies will be introduced throughout the year-long curriculum and focused especially in this first in-person Short Course. Faculty and technical team members (mentors) share expertise in these ICT areas and will draw upon one another when providing scheduled (and as needed) consultation and mentoring for the teams. Team mentors will assemble annually in Rochester and in Costa Rica to share new approaches, technological ideas, relevant advances in global health and biomedical research, and to continually improve program infrastructure and resources. Throughout the program, teams will collaborate with the mentors to develop their basic ICT intervention which addresses their identified maternal health issue.

### ICT in maternal health II short course: communications, evaluation

During the second short course (Table 3) the teams are expected to have refined their projects and evaluation plans, including new ICT technologies and applications for future work. Teams will generate collaborative research ideas with faculty and technical advisors for future funding strategies. Additionally, they will learn to use global health ICT resources beyond Latin America.

The evaluation of online training and mentoring included longer-term outcomes (evaluation of Moodle® platform experience) and shorter-term outcomes: whether ICT innovations were developed and tested for feasibility in community-based real-life settings, whether the training program infused each institution and each of the trainee teams with new insights and strategies for working with diverse partners to create ICTs, whether the Collaboratory concept of mentoring and social networking aided the teams and faculty to improve the efficiency of the development process, and whether the academic collaboration amongst the partners strengthened. These outcomes directly relate to the project’s four primary aims, as described above.

#### Evaluation of the short course and Moodle® online platform

Using information technology for assessment (e-assessment) includes various strategies to evaluate trainee learning [20–22]. e-Assessment can be accomplished within a single-user system, via networks linked to specific servers, or directly online [23]. As with any assessment, e-assessment provides feedback to trainees about their progress and about whether they have met learning objectives [20].

A pre-training questionnaire and daily post-training evaluations were piloted with the 12 trainees in the first year, using a range of ratings and response choices. A post-training questionnaire was later provided to the first cohort prior to the beginning of their second face-to-face training, to assess the longer term online Moodle® platform experience.

## Results

Trainees were assessed for knowledge and familiarity prior to the face-to-face workshop, and daily after each day’s educational workshop activity. Baseline evaluation of the trainees (*n* = 8) indicated gaps in knowledge of ICTs (Mean: 4.7/10; Range: 2.8/10 to 6.0/10), with the highest familiarity reported for social networking. Although multiple reminders and encouragement were given to all 12 trainees, only six anonymously participated in immediate post-training assessments. Average scores indicated self-reported increases in knowledge across course content areas (see Table 4). A total of 10 participants replied to the post-training online Moodle® experience evaluation (see below).

**Table 4:** Baseline and Immediate Post-Training Average Scores

Evaluation of Course Topics	Average Scores Scale 1-10, where: 1 = Knows a Little 10 = Knows a Lot
Baseline knowledge of course topics	4.7**
Prepared for Online Moodle® Modules	5.8*
Prepared for using REDCap	6.7*
Cloud Computing	6.8*
Word Press/HTML	8.3*
Other software/technology	8.3*
Social Networks and Community Empowerment	8.3*

*n = 6; **n = 8

Likert Scales were either 1–4 or 1–5. For purposes of comparison across educational topics, all scores were all standardized to scores between 1 and 10

With respect to social media, the pre-training feedback indicated that trainees felt particularly competent in the understanding and use of various social media. At the end of the in-person, five day course, however, anecdotal feedback in a group discussion indicated that they felt their self-reported pre-training knowledge did not match with what they learned during the week. For example, they thought they understood social media well, but learned that social media can be used not just for one’s own social interactions and entertainment, but as a means of reaching their target populations and delivering a variety of interventions.

Feedback was also elicited on didactic presentations, on specific technologies and social networks (including Google Cloud®, Amazon Web Services® (AWS®), Twitter®, Facebook®, etc.), distance learning platforms (e.g., Moodle®), and on public health models. Daily feedback was also elicited for specific presenters and events relevant to that day. For example, at the end of one of the training days, all of the trainees either “agreed” or “strongly agreed” with the statement: “*Presentaron información actual y parecieron bien informados sobre el tema* (They presented actual information and appeared to be well informed on the topics discussed).”

Other open-ended questions elicited specific feedback. For example:

**Table:** 

**Spanish**	**English**
*Muy interesante el uso de aplicaciones para la implementación de nuestros proyectos*	The use of applications for implementation of our projects was very interesting
*Es muy interesante todo lo que han explicado para mi*	Everything that they have explained to me was very interesting
*Proveer más ejemplares sobre el primer tema*	Provide more examples for the first theme
*El desarrollo del curso fue muy interesante y he aprendido mucho*	The course development was very interesting and I learned a lot

Post-training evaluation of the online Moodle® course included the observations that 1) students completed online course assignments with prompts and encouragement from mentors as needed, and 2) at the end of the online coursework participants responded to overall evaluation questions of the online MOODLE® experience (see Tables 5 and 6).

**Table 5:** Online Moodle® Course Evaluation Items*

	Neither Agree		
	Agree	Nor Disagree	Disagree
In general, I was satisfied with the online Moodle® course.	*n* = 7 (70%)	*n* = 1 (10%)	*n* = 2 (20%)
I learned new things from this Moodle® course	*n* = 9 (90%)	*n* = 0 (0%)	*n* = 1 (10%)
I am confident that I can use the things I have learned in the Moodle® course	*n* = 9 (90%)	*n* = 0 (0%)	*n* = 1 (10%)
This Moodle® course teaches me important themes	*n* = 8 (80%)	*n* = 1 (10%)	*n* = 1 (10%)
I would recommend this Moodle® course to others	*n* = 7 (70%)	*n* = 2 (20%)	*n* = 1 (10%)

* *n* = 10. All questions and answers were in Spanish. Responses, translated to English, are included here with corresponding subject “n’s” and percentage endorsements of Strongly Agree, Neither Agree Nor Disagree, Disagree, and Strongly Disagree. Strongly Agree and Agree were collapsed for this table and presented as “Agree”. Strongly Disagree and Disagree were collapsed for this table and presented as “Disagree”

**Table 6:** Open Ended Evaluation Questions*

Questions	Common Themes	Example Quotes
What are the 3 most important things you learned from the Moodle® course?	Crowdsourcing	How crowdsourcing could help us with the evaluation and users’ information on a research study
	ICTs (Health Related)	The importance of the use of ICT’s in maternal health)
	Building a Logic Model	How to design a logic model
What could be improved for this Moodle® Course?	Moodle® platform	The access to upload or to send assignments should be less complicated to students
	Language difficulties	Some pages were only in English
Do you have any other comments for this Moodle® course?	Courses and Research	I think these types of courses should always be available, they increase the knowledge and help to resolve problems according to the topics or projects that can be investigated

* *n* = 10. All questions and answers were in Spanish. Themes were identified for inclusion when at least two respondents endorsed a particular theme. Exemplary respondent quotes, translated to English, are included here

## Discussion

MundoComm aims to address the primary issue of maternal health problems in Costa Rica and other LMICs in Latin America by addressing existing knowledge and practice gaps among its partners, and to improve the technological capacity of their community work and public health research to integrate real-world, significant problems, and for communities to become familiar with the innovation process and technological resources it can access to address its pressing problems.

The course and modules were developed to include best practices in Information and Communication Technologies (ICT) and e-Health strategies, appropriate ethical review and institutional involvement, proactive community engagement, and community interventions that are evidence-based. Evaluations (in-person and online) involve both quantitative and qualitative feedback. For example, to address “community engagement”, a module specifically addressed maternal health data, community engagement, and project organization via assignments such as comparing their own country’s data with data from other countries, how to conduct semi-structured qualitative interviews, and developing logic models.

ICT technologies have emerged rapidly within maternal and child global health communities [8, 9], and growing evidence indicates that ICT interventions can significantly improve maternal health in lower- and middle-income countries [24, 25]. For example, implementing basic perinatal registries to track maternal health patterns using analytics helps communities better identify effective strategies, track outcomes, and target high-risk areas [26, 27], while continually improving data quality [28]. The use of electronic health records, such as electronic perinatal medical records, have demonstrated maternal health improvement in under-resourced areas of the United States [29] and globally [30, 31].

Websites form an important mechanism for dissemination of health information to women [32] whose usage differs from that of men [33], and can deliver effective wide-reaching interventions such as tobacco cessation [34, 35], and other risk behavior changes that are strongly linked to maternal health outcomes. Relatedly, social media connect pregnant and post-partum women to each other, and research shows this social media engagement may improve well-being [33], management of pregnancy-associated weight and nutrition [36]. Finally, ICT interventions need to be tailored with women’s usability in mind, since research has shown post-partum women favor websites that have easy navigation, and that promote social interaction with others [37].

MundoComm therefore focuses on basic training across several technical areas of ICT (prioritizing Open Access and free tools): Databases (REDCap®) and Analytics (EpiInfo®); Electronic Health Records (e.g., electronic perinatal medical records); HTML/Web Site Design (WordPress®); Mobile App development (PhoneGap®, OpenMEAP®); and Social Media (Facebook®, Twitter®, LinkedIn®, Google Analytics®). As “Cloud Computing” may provide a paradigm shift in how low-resource communities may access and use a wide range of tools and technical details [38], the training will also introduce such Cloud Computing options as OwnCloud®, Amazon Web Services®, and Google Cloud®. Beyond the current project, the long-term impact is expected to include development and scale-up of ICT interventions that improve maternal health, in the sustained, collaborative context of multidisciplinary stakeholders. Online tools used in MundoComm were selected in part with consideration to being user-friendly, with various interactive features, and maximum flexibility. For example, REDCap® was used for a variety of purposes, including initial team applications, uploading documents, and evaluations of face-to-face and online modules. This tool can be accessed and used for data capture from a variety of devices, both online and offline [39].

With respect to MundoComm’s four aims, described above, the current program has already demonstrated 1) the feasibility of implementing a mentored training program for community-based public health applied research teams in LMICs, with specific reference to maternal health; 2) the implementation of specific training team ICT innovations in field settings (resulting in the eventual completion of each team’s proposed ICT project); 3) the creation of a “Collaboratory” environment where teams, faculty, experts, and others to share developing material and processes; and 4) the promising development of a professional network of ICT, medical, public health, and community students, faculty, researchers, and practitioners devoted to supporting ICT for maternal health in LMICs. If ongoing evaluation in this and related projects can provide a “proof of concept”, the present mixed model of training with the use of technology (i.e. online courses for distance learning and mentoring learners with only 2 weeks of face to face contact) is potentially feasible and sustainable in LMICs.

As described by Heller et al. [1], in the context of education, students are not just recipients but are actively involved in collaboration in learning activities, often expressed as Web 2.0, eLearning 2.0 or Education 2.0. The emergence of Education 3.0 [40], they posit, is considered to be an extension of this, where open-access materials are created and adapted by various collaborating groups and individuals – including the students. These thoughts are consonant with MundoComm’s four primary aims, particularly with respect to the creation of a “Collaboratory”, and with the incorporation of feedback from trainees as part of our iterative curricular development.

### Challenges

Maximizing novel approaches to online training and program management both in the US and country-specific requires experimentation with sophisticated technologies including multi-media presentation of didactic material, distance learning strategies, and the use of iPads for offline data collection in global settings. Although comparing and selecting optimal media, strategies, software and hardware would have been ideal for enhanced project fidelity; this was not fully possible due to the project’s timeline and available resources.

Although useful as a qualitative assessment of feedback from this first cohort, the Likert style items will be refined and standardized for future cohorts, incorporating trainee feedback. These will be administered to subsequent cohorts to quantitatively assess measurable pre-post changes in self-rated scores. For the first cohort however, where Likert ranges included both 1–4 and 1–5 response choices, standardized proportional scores (1–10) were calculated from average scores for simple comparison purposes.

### Lessons learned

For faculty there was a steep learning curve, initially, as the online platform had parallel but different features than other LMS systems used previously – coupled with the fact that initial instruction on the use of Moodle® was in Spanish only, and based on the host institution’s online virtual environment/version of Moodle®. Not unexpectedly, there were challenges to creating, implementing, and tracking online systems for both educational and data collection purposes. Preliminary evaluation data indicate overall satisfaction with the course format (both in-person short courses, and online didactic components) and the content presented.

### Future directions

The second in-person short course will come in the ninth month of the training program, approximately 5 months after the first short course, described above. By that time, the trainee teams will be entering the last phase of their intervention development. The second short course will be focused more on bringing team projects toward closure and evaluation, and introducing new concepts and technologies. It will also feature more time with mentors and advisors in multiple interactive workshop settings, with a focus on resolving details and expanding on project ideas. Online training will continue with the six modules described above, and will overlap with this second in-person short course. Both the latter online modules and the second short course will continue to be informed by our ongoing iterative curricular developmental process involving evaluation feedback from trainees, project staff, and other stakeholders. The final outcome (self-reported community involvement, project pilot development, etc.), will be measured 1 year after the end of the second short course. Given the flexibility and availability of our primary course platforms and tools (e.g., Moodle® and REDCap®), future courses can be expanded upon and/or scaled up as appropriate. Such tools can easily accommodate multiple designers, didactic tools (including an increasing variety of multi-media options), and evaluation strategies.

As the aims of MundoComm are to train and demonstrate the successful use of ICTs for maternal health improvement in LMICs, evaluation efforts at the conclusion of the training will assess trainees’ perceived learning of evidence-based strategies to decrease maternal health disparities and the degree of incorporation of valid evaluation strategies to assess the impact of each project’s initiative on appropriate measurable health outcomes.

Determinants of the reproducibility of the present model include start-up costs and the availability of online technological resources. Costs in the present model can be minimal, as there are advantages to using existing online coursework platforms through partnering institutions (MOODLE®, REDCap®), although time was needed for communicating across international teams to address issues related to troubleshooting and access to training models for all faculty, mentors, and trainees. Programs should establish a stable and accessible learning platform and project teams should be able to have ongoing reliable access to the internet.

Consistent with the tenets of CBPR (Community Based Participatory Research) and of our previous work [4–6, 11], there are a number of factors that all serve to increase the likelihood of community uptake and the reliability of transporting infrastructure such as the present model more broadly. These include: 1) the use of field teams embedded in communities in which their projects were developed, 2) team selection based on a maternal health topic relevant to their community, and 3) community engagement supported throughout the process of project development.

### Conclusion

Initial evaluations and feedback during the implementation phase suggest that, in combination with in-person learning, an online training platform and e-assessment strategies can be feasible for delivering targeted didactic material. The present study preliminarily evaluates feasibility, usability and progress towards MundoComm’s aims and learning objectives. The advantages to the type of online strategies employed, such as striving for usability, cultural appropriateness, and the incorporation of a variety of interactive features to enhance the learning experience, can help inform future initiatives towards maximizing flexibility in training. With ongoing feedback from all stakeholders, including trainees, additional functionality and efficacy can be pursued. Lessons learned from this training experience include the development of better practices to ensure more complete evaluations, such as assigning unique user identification credentials to participants to anonymously track their participation and secure evaluation feedback.

Our multidisciplinary approach to training with face-to-face didactics and distance learning strategies resulted in the implementation of a model-based course for training maternal health professionals, the process of which can potentially be exportable to other settings. Newer interfaces for online training will help us fully optimize e-learning as a mode of delivery for global learning.

The community-engagement process links teams with their primary stakeholders, communities and institutions [14]. By involving community perspectives into creative ideation, project design, and revision, team members are facilitating accountability for completing their projects, with the encouragement and involvement of the community. When institutions and communities are aware of, supportive of, and involved in a team’s work, teams are even more likely to be motivated to complete the work and be accountable for its outcome. Additionally, all team materials are housed and shared within the Collaboratory virtual space, such that others from outside MundoComm and new trainees within it can access them. This open-source perspective further fosters a sense of collaborative purpose and shared vision.

Finally, trainees will emerge as thought-leaders who are knowledgeable around issues of ICT in maternal health and, to a broader degree, public health. These individuals are likely to continue their relationships with their mentors and remain involved with MundoComm through its social network of professionals and stakeholders. Given the level and role of trainees in their institutions and in their communities, investing in their training and promoting research partnerships with the major institutions of medicine and technology involved with this project will enhance the project’s ability to continue momentum in the future.

## Abbreviations

AIT: Academic Information Technology
AWS: Amazon Web Services®
CBPR: Community Based Participatory Research
CER: Community-Engaged Research
CTSI: Clinical Translational Science Institute
GNLE: Globally networked learning environment
HIPAA: Health Insurance Portability and Accountability
HTML: Hypertext markup language
ICT: Information and communication technology
IRB: Institutional review bard
IT: Information technology
LMIC: Low and middle income countries
LMS: Learning management systems
MOODLE®: Modular Object-Oriented Dynamic Learning Environment
MundoComm: Training initiative funded by the National Institutes of Health
NIH: National Institutes of Health
REDCap®: Research Electronic Data Capture
RSRB: Research Subjects Review Board
TLT: Team Learning Theory
UCIMED: Universidad de Ciencias Médicas
US: United States
